# Assessing health state utilities for people with myalgic encephalomyelitis/chronic fatigue syndrome in Australia using the EQ-5D-5L, AQoL-8D and EQ-5D-5L-psychosocial instruments

**DOI:** 10.1007/s11136-023-03498-8

**Published:** 2023-08-10

**Authors:** Nneka C. Orji, Ingrid A. Cox, Leonard A. Jason, Gang Chen, Ting Zhao, Melissa J. Rogerson, Ryan M. Kelly, Karen Wills, Martin Hensher, Andrew J. Palmer, Barbara de Graaff, Julie A. Campbell

**Affiliations:** 1 Menzies Institute for Medical Research, University of Tasmania, Hobart, TAS Australia; 2 Centre for Health Economics, Monash University, Melbourne, VIC Australia; 3 School of Population and Global Health, The University of Melbourne, Melbourne, VIC Australia; 4 DePaul University Center for Community Research, Chicago, IL USA; 5 School of Computing & Information Systems, University of Melbourne, Melbourne, VIC Australia

**Keywords:** Quality of life, ME, CFS, Health-related quality of life, Health state utility, Psychosocial health

## Abstract

**Purpose:**

Myalgic encephalomyelitis/chronic fatigue syndrome (ME/CFS) is a chronic condition with a constellation of symptoms presenting as severe and profound fatigue of ≥ 6 months not relieved by rest. ME/CFS affects health-related quality of life (HRQoL), which can be measured using multi-attribute health state utility (HSU) instruments. The aims of this study were to quantify HSUs for people living with ME/CFS, and to identify an instrument that is preferentially sensitive for ME/CFS.

**Methods:**

Cross-sectional national survey of people with ME/CFS using the AQoL-8D and EQ-5D-5L. Additional questions from the AQoL-8D were used as ‘bolt-ons’ to the EQ-5D-5L (i.e., EQ-5D-5L-Psychosocial). Disability and fatigue severity were assessed using the De Paul Symptom Questionnaire-Short Form (DSQ-SF). HSUs were generated using Australian tariffs. Mean HSUs were stratified for sociodemographic and clinical factors. Bland–Altman plots were used to compare the three HSU instruments.

**Results:**

For the 198 participants, mean HSUs (95% confidence intervals) were EQ-5D-5L: 0.46 (0.42–0.50); AQoL-8D: 0.43 (0.41–0.45); EQ-5D-5L-Psychosocial: 0.44 (0.42–0.46). HSUs were substantially lower than population norms: EQ-5D-5L: 0.89; AQoL-8D: 0.77. As disability and fatigue severity increased, HSUs decreased in all three instruments. Bland–Altman plots revealed interchangeability between the AQoL-8D and EQ-5D-5LPsychosocial. Floor and ceiling effects of 13.5% and 2.5% respectively were observed for the EQ-5D-5L instrument only.

**Conclusions:**

ME/CFS has a profound impact on HRQoL. The AQoL-8D and EQ-5D-5L-Psychosocial can be used interchangeably: the latter represents a reduced participant burden.

**Supplementary Information:**

The online version contains supplementary material available at 10.1007/s11136-023-03498-8.

## Introduction

### Myalgic encephalomyelitis/chronic fatigue syndrome

Myalgic encephalomyelitis/chronic fatigue syndrome (ME/CFS) is a chronic, multisystemic disease with complex manifestations [1, 2]. Its clinical manifestations are heterogenous in nature with an identifiable pattern; however, it generally presents with disabling extreme post-exertional malaise (PEM) [2]. PEM is the pathological inability of the body system to generate adequate energy on demand: a massive and prolonged energy deficit which results in the inability to perform normal and routine activities [2]. PEM usually arises in concert with other disabling and prolonged systemic manifestations such as unrefreshing sleep, orthostatic intolerance and cognitive impairment [3, 4]. The chronic nature of ME/CFS affects the work productivity and health-related quality of life (HRQoL) of people living with the condition over time [5–8]. Furthermore, ME/CFS affects more persons from the age groups most likely to be in the workforce [5, 6, 9].

### Health state utilities as a measure of health-related quality of life

Health state utilities (HSU) are metrics that measure the strength of preference for a particular health state, represented as a number between 0 and 1, where 0 is anchored to death (or health states equivalent to being dead) and 1 corresponds to ideal health [10–12]. Health states worse than death are possible, represented by negative HSU [13]. HSU are used in cost-utility analyses (CUA) for Health Technology Assessments [11]. There are several approaches to deriving HSU, with multi-attribute utility instruments (MAUI) commonly used [11]. As well as being an input metric to CUA, HSU have also been shown to be independent predictors of patient outcomes, including all-cause mortality and development of complications [14]. Moreover, clinicians have found that measuring HRQoL and HSU is beneficial to patients regarding clinical assessment, relationships, communication, and management [15].

### HSU for people with ME/CFS

Just three studies assessing HSU for people living with ME/CFS have been published, originating from the UK [16, 17] and Denmark [8]. Overall, mean HSU were consistently low, ranging between 0.36 and 0.56 using the EQ-5D-3L [8, 16, 17] instrument. However, to the best of our knowledge there are no studies that assess HSU against disease severity classifications for ME/CFS, hence assessing cost-utilities for ME/CFS remains challenging.

Some studies have used generic HRQoL measures that are not MAUI (and therefore do not measure HSU) such as the SF-36 [7] or World Health Organization Quality of Life questionnaire (WHOQOL-BREF) [18] to reveal diminished HRQoL for people living with ME/CFS. An Australian study examined the impact of sociodemographic and patient symptoms using the SF-36 and reported significantly lower scores across all domains compared to the general population [7]. Similarly, another Australian study explored HRQoL (physical functioning and psychological distress) for people living with ME/CFS and reported profound effects on physical functioning in addition to increased manifestations of psychological distress [19]. Another much older Australian study (1995) used the Sickness Impact Profile and patient interviews to demonstrate that ME/CFS had a significant impact on the quality of life of people with ME/CFS, especially on their social functioning [20]. A multi-country study reported similarly reduced HRQoL for people living with ME/CFS [21].

### Aims of this study

The primary aim of this study was to investigate HSU for people with ME/CFS. In addition, we aimed to identify a MAUI with a propensity for the physical and/or psychosocial domains of health and that is preferentially sensitive for Australians with ME/CFS.

## Methods

### Survey of people living with ME/CFS

A national survey using convenience sampling was conducted between August and December 2021 to assess the human and economic impacts of ME/CFS. To inform the development of a tailored questionnaire, we conducted focus groups with people living with ME/CFS. Based on these, we included questions about costs incurred due to ME/CFS: costs related to electronic and digital equipment and ‘apps’, childcare, everyday living (e.g., cleaning, gardening services), specialised home modification or renovations, and special dietary requirements. Furthermore, we worked closely with the Patient Advisory Group to ensure the survey captured relevant information and was delivered in such a way as to reduce cognitive burden on participants. The online survey was hosted using the Qualtrics platform in modules with an estimated completion time presented for each. This was done so participants could pace themselves and complete modules during separate sessions. The survey included questions on disability and fatigue severity using the de Paul Symptom Questionnaire-Short Form (DSQ-SF) [22], and on co-morbidities (including diabetes, cardiovascular disease and mental health and classified as 0, 1, 2 and 3 or more co-morbidities). HSU were assessed using the AQoL-8D, EQ-5D-5L and EQ-5D-5L-Psychosocial instruments. To minimize bias that may arise from asking repetitive questions on HRQoL, the EQ-5D-5L and AQoL-8D instruments were delivered to participants in random order, with 50% completing the EQ-5D-5L first and 50% completing the AQoL-8D first. Ethics approval was granted by the University of Tasmania’s Health and Medical Research Ethics Committee (H0018473).

### Eligibility criteria and recruitment

Eligibility criteria for our participants included a self-reported diagnosis of ME/CFS and aged 18 years or older and living in Australia. Participants were recruited through newspaper advertisements, newsletters, and social media posts of Emerge Australia (a national organisation providing education, advocacy, research, and support services).

### The instruments: EQ-5D-5L, AQoL-8D and EQ-5D-5L-psychosocial

Table 1 shows the characteristics of the three MAUI. The EQ‐5D-5L asks participants to indicate whether they have problems on a five-level scale for each of the five dimensions of health: mobility, self‐care, usual activities, pain/discomfort, and anxiety/depression. The EQ-5D-5L was developed to address the limited sensitivity (lack of descriptive richness and serious ceiling effects) of its predecessor the EQ-5D-3L [23] and describes 3125 health states. The algorithmic range for most of the instrument’s country-specific value sets describes HSU ranging from < 0 to 1.0[13].

**Table 1:** Comparison of the key characteristics of the EQ-5D-5L, AQoL-8D and EQ-5D-5L-Psychosocial multi-attribute utility instruments and the Short Form De Paul Questionnaire

	EQ-5D-5L	AQoL-8D	EQ-5D-5LPsychosocial	Short Form De Paul Questionnaire
Dimensions	Five dimensions: Mobility Pain/discomfort Depression/anxiety Self-care Usual activities	Eight individual dimensions: Mental health Coping Happiness Relationships Self-worth Pain Independent living Senses Two super-dimensions: Psychosocial super-dimension Physical super-dimension	Nine dimensions: *EQ-5D-5L:* Mobility Pain/discomfort Depression/anxiety Self-care Usual activities and *Four AQoL-8D bolt-ons:* Vitality Sleep Community Connectedness Relationships	Eight dimensions: Fatigue Post exertional malaise Sleep Pain Neurocognitive Autonomic Neuroendocrine Immune
Items	5	35	9	54
Response levels	5	4–6	4–6	5
Health states	3125	2.4 × 10^23^	1,953,125	Not applicable
Measurement unit	Health state utility	Health state utility, super-dimensions (2), and individual dimensions (8)	Health state utility	Frequency and severity rating
Theoretical scale	0–1.00 0 worst score/death 1 full health Scores at less than 0 indicate health states worse than death	0–1.00 0 worst score/death 1 full health	0–1.00 0 worst score/death 1 full health	5-point Likert scale 0–4
Theoretical utility range	− 0.59 to 1.00	0.06–1.00	0.06–1.00	Not Applicable
Missing values	Not allowed	Up to 10	Not allowed	Not allowed

The AQoL-8D was originally developed to achieve sensitivity not only in health states affected by physical disorders, but also in those affected by mental disorders [24]. This instrument contains 35 items in eight dimensions and was derived using psychometric methods for achieving content validity. Three of the dimensions (independent living, pain, senses) load to a physical super-dimension; the other five (mental health, happiness, coping, relationships, and self-worth) load to a mental super-dimension. The size of the instrument means that it can define billions of health states [24].

The new EQ-5D-5L-Psychosocial was developed by Chen and Olsen in 2020 [25] and was externally validated for a large cohort with multiple sclerosis (MS) [26]. It was developed to address the psychosocial deficiencies of the EQ-5D-5L by including four additional bolt-on dimensions of vitality, relationships, sleep and community connectedness, adopted from the AQoL-8D (Table 1) [25]. The developmental phase of this new instrument revealed that vitality was the most important dimension with regard to HRQoL [25]. Given the dominant position of the EQ-5D-5L in applied studies, the developers suggested that identifying a set of bolt-on dimensions that captured the psychosocial aspects of health would serve as a realistic alternative for developing a completely new extended generic preference-based measure [25]. The scoring algorithm was developed from a mapping analysis that mapped responses to nine items (five EQ-5D-5L and four bolt-on items) onto the AQoL-8D utilities.

Australian population norms for the AQoL-8D and EQ-5D-5L were sourced from literature to correspond to the mean age of our study population i.e. norm for the AQoL-8D is 0.77 [27] and for the EQ-5D-5L is 0.89 utility points [28].

### Disability and fatigue severity classifications

Table 1 shows the characteristics of the DSQ-SF [29]. It consists of 14 questions (with two 5-point Likert scales for each question) and has been validated against the Canadian Consensus Criteria [2]. Disability severity was calculated using the DSQ-SF. We adopted a validated methodology used in a previous work on multiple sclerosis to map from the Patient Determined Disease Steps (9 questions regarding gait) to the Expanded Disability Status Scale [30]. Following the logic of this disability severity classification, we calculated disability severity for each individual who entered the study by assigning values of no disability (0), mild disability (1), moderate disability (2) and severe disability (3) to the five Likert responses (with increasing disability) to the 28 DSQ-SF questions with 0 = 1; 1 = 2; 2 = 3; and 3 = 4 and 5 for each question and then calculating the average across the 14 questions. The final disability severity score used the cut points of < 0–0.5 (no disability); 0.6–1.5 (mild disability); 1.6–2.5 (moderate disability); and > 2.5 (severe disability). Fatigue severity was classified by selecting seven questions directly related to fatigue symptoms such as fatigue, tiredness after minimal exercise and unrefreshing sleep, and followed the same method to select fatigue severity questions (Supplementary Table 1).

### Statistical methods

Summary statistics describing the baseline sociodemographic and clinical characteristics (Table 2) of participants were generated and presented as mean and standard deviation (SD) or median and interquartile range (IQR) (Supplementary Table 3) for continuous variables, and frequency counts and percentages for categorical variables (Table 3). Completion rates for all three MAUI were assessed and summarized. HSU were described using frequency distributions and summary statistics (mean, 95% CI and range). HSU were generated using the Australian tariffs [13, 25, 31] noting that there can be 10 missing patient-reported responses for the AQoL-8D, and no missing values for the EQ-5D instruments. An Australian value set for the EQ-5D-5L has not been published to-date; therefore we used an Australian algorithm based on a discrete choice experiment [13]. The algorithm for the EQ-5D-5L-Psychosocial (using Australian data) was adopted [25]. Histograms were used to visualize the frequency distribution of the individual HSU in each MAUI (Supplementary Fig. 2)**.**

**Table 2:** Sociodemographic and clinical characteristics of participants

Participant characteristics (*n* = 198)	
*Sociodemographic*	
Age (years)	
Mean (SD)	48.7 (14.3)
Age category, years	*n* = 197 (%)
< 45	73 (37.1)
46–84	124 (62.9)
Gender	*n* = 198 (%)
Male	32 (16.2)
Female	158 (79.8)
Other	8 (4.0)
Education	*n* = 197 (%)
≤ Year 12	25 (12.7)
Trade*	40 (20.3)
Bachelors	65 (33.0)
Postgraduate	67 (34.0)
Employment	*n* = 172 (%)
Too unwell to be employed	80 (46.5)
Unemployed	n/r
Retired	19 (11.1)
Full time	12 (7.0)
Part time	46 (26.7)
Student	6 (3.5)
Other	6 (3.5)
Income ($AUD)	*n* = 195 (%)
Nil/negative income	25 (12.8)
< 400/week	51 (26.2)
400–799/week	79 (40.5)
800–1249/week	17 (8.7)
≥ 1250/week	23 (11.8)
Marital status	*n* = 198 (%)
Married/De facto	87 (43.9)
Divorced/separated	30 (15.2)
Single	77 (38.9)
Widowed	n/r
Geographical remoteness*	*n* = 192 (%)
Major capital city	113 (58.9)
Inner regional	54 (28.1)
Outer regional	25 (13.0)
*Clinical*	
Disability severity	*n* = 198 (%)
No	n/r
Mild	40 (20.2)
Moderate	137 (69.2)
Severe	19 (9.6)
Fatigue severity	*n* = 198 (%)
No	n/r
Mild	22 (11.1)
Moderate	118 (59.6)
Severe	56 (28.3)
Comorbidities *n* (%)	*n* = 198 (%)
0	128 (64.7)
1	48 (24.2)
2	18 (9.1)
3	n/r

Trade = trade certificate or diploma , n/r = cell sizes < 5 not reported to maintain participant anonymity

Missing data: age category = 1; gender = 1; education = 1; employment = 26; income = 6; jurisdiction = 6; geographical remoteness = 6

*Geographic remoteness based on ABS Geographic Statistical Areas [41]

**Table 3:** Summary statistics of HSUs stratified by sociodemographic and clinical characteristics for the EQ-5D-5L, AQoL-8D and EQ-5D-5L-Psychosocial instruments

	EQ-5D-5L		AQoL-8D		EQ-5D-5L-Psychosocial	
	Mean (95% CI)	Range	Mean (95% CI)	Range	Mean (95% CI)	Range
Overall sample	0.46 (0.42−0.50)	− 0.50–1.00	0.44 (0.42–0.45)	0.15–0.99	0.44 (0.42–0.46)	0.11–0.98
*Sociodemographic*						
Age group (years)						
< 45	0.37 (0.30–0.44)	− 0.43–0.86	0.41 (0.37–0.43)	0.15–0.76	0.41 (0.38–0.44)	0.12–0.78
46–84	0.51 (0.45–0.57)	− 0.50–1.00	0.46 (0.41–0.49)	0.16–0.99	0.46 (0.43–0.49)	0.11–0.91
Gender						
Male	0.53 (0.44–0.62)	− 0.11–1.00	0.47 (0.40–0.54)	0.16–0.99	0.46 (0.40–0.52)	0.14–0.91
Female	0.45 (0.40–0.50)	− 0.50–1.00	0.44 (0.42–0.46)	0.15–0.99	0.44 (0.42–0.46)	0.11–0.98
Other	0.35 (− 0.01 to 0.71)	− 0.43–0.81	0.38 (0.26–0.50)	0.17–0.64	0.38 (0.25–0.51)	0.15–0.59
Education level						
≤ Year 12	0.44 (0.27–0.61)	− 0.43–1.00	0.42 (0.35–0.49)	0.17–0.99	0.42 (0.35–0.49)	0.15–0.98
Trade	0.39 (0.30–0.48)	− 0.17–0.81	0.40 (0.36–0.44)	0.15–0.74	0.39 (0.35–0.43)	0.12–0.65
Bachelors	0.46 (0.38–0.54)	− 0.50–1.00	0.44 (0.41–0.47)	0.16–0.82	0.45 (0.42–0.48)	0.11–0.75
Postgraduate	0.51 (0.44–0.58)	− 0.19–1.00	0.48 (0.44–0. 52)	0.20–0.99	0.48 (0.44–0.52)	0.16–0.91
Employment						
Unwell	0.40 (0.33–0.47)	− 0.43–1.00	0.40 (0.37–0.43)	0.16–0.76	0.40 (0.37–0.43)	0.12–0.68
Unemployed	0.46 (0.06–0.86)	0.34–0.64	0.45 (− 0.07 to 0.97)	0.28–0.68	0.43 (0.13–0.73)	0.33–0.56
Retired	0.63 (0.50–0.76)	− 0.13–0.92	0.57 (0.48–0.66)	0.25–0.87	0.56 (0.49–0.63)	0.27–0.75
Full-time	0.70 (0.60–0.80)	0.49–0.92	0.55 (0.46–0.64)	0.33–0.78	0.55 (0.46–0.64)	0.28–0.78
Part-time	0.51 (0.43–0.59)	− 0.06–1.00	0.46 (0.42–0.50)	0.15–0.99	0.45 (0.41–0.49)	0.12–0.91
Student	0.52 (0.31–0.73)	0.32–0.81	0.45 (0.31–0.59)	0.29–0.64	0.42 (0.30–0.54)	0.29–0.59
Other	0.62 (0.55–0.69)	0.49–0.67	0.46 (0.36–0.54)	0.32–0.59	0.49 (0.42–0.56)	0.34–0.54
Income						
Nil/negative income	0.45 (0.32–0.58)	− 0.11–1.00	0.47 (0.38–0.54)	0.23–0.99	0.45 (0.37–0.53)	0.14–0.98
≤ 399/week	0.42 (0.32–0.52)	− 0.50–1.00	0.39 (0.34–0.42)	0.16–0.76	0.40 (0.36–0.44)	0.11–0.69
400–799/week	0.42 (0.35–0.49)	− 0.42–1.00	0.43 (0.39–0.45)	0.16–0.76	0.43 (0.40–0.46)	0.14–0.69
800–1249/week	0.53 (0.39–0.67)	− 0.17–0.92	0.50 (0.39–0.57)	0.28–0.87	0.48 (0.40–0.56)	0.18–0.75
≥ 1250/week	0.63 (0.53–0.73)	< − 0.01–1.00	0.52 (0.42–0. 58)	0.15–0.99	0.51 (0.44–0.58)	0.12–0.91
Marital status						
Single	0.36 (0.28–0.44)	− 0.43–1.00	0.41 (0.38–0.44)	0.15–0.99	0.40 (0.37–0.43)	0.12–0.98
Married	0.53 (0.47–0.59)	− 0.50–1.00	0.47 (0.44–0.50)	0.16–0.87	0.47 (0.44–0.50)	0.11–0.78
Divorced/separated	0.47 (0.36–0.58)	− 0.08–0.92	0.43 (0.38–0.48)	0.20–0.68	0.43 (0.37–0.49)	0.12–0.73
Widowed	0.69 (0.29–1.00)	0.39 to 1.00	0.64 (0.19–1.00)	0.40–0.99	0.60 (0.23–0.97)	0.40–0.91
Geographical remoteness area						
Major city	0.45 (0.39–0.51)	− 0.50–1.00	0.44 (0.41–0.47)	0.16–0.99	0.44 (0.41–0.47)	0.11–0.91
Inner regional	0.49 (0.41–0.57)	− 0.24–1.00	0.45 (0.41–0.49)	0.15–0.99	0.44 (0.40–0.48)	0.12–0.98
Outer regional	0.45 (0.31–0.59)	− 0.28–1.00	0.46 (0.31–0.51)	0.25–0.74	0.44 (0.38–0.50)	0.14–0.65
*Clinical*						
Disability severity						
No	1.00 (1.00–1.00)	1.00–1.00	0.99 (0.90–1.00)	0.99–0.99	0.94 (0.49–1.00)	0.91–0.98
Mild	0.69 (0.63–0.75)	− 0.11–1.00	0.57 (0.53–0.61)	0.27–0.87	0.55 (0.51–0.59)	0.29–0.75
Moderate	0.44 (0.39–0.49)	− 0.43–1.00	0.42 (0.40–0.44)	0.16–0.76	0.43 (0.41–0.45)	0.14–0.78
Severe	0.02 (− 0.13–0.17)	− 0.50–0.63	0.30 (0.25–0.35)	0.15–0.55	0.25 (0.20–0.30)	0.11–0.46
Fatigue severity						
No	1.00 (1.00–1.00)	1.00–1.00	0.99 (0.90–1.00)	0.99–0.99	0.94 (0.49–1.00)	0.91–0.98
Mild	0.69 (0.62–0.78)	0.33–1.00	0.61 (0.54–0.68)	0.27–0.87	0.57 (0.52–0.62)	0.29–0.75
Moderate	0.55 (0.51–0.59)	− 0.17–1.00	0.45 (0.43–0.47)	0.16–0.76	0.47 (0.45–0.49)	0.14–0.78
Severe	0.16 (0.08–0.24)	− 0.50–0.67	0.34 (0.31–0.37)	0.15–0.60	0.31 (0.28–0.34)	0.11–0.56
Comorbidities						
0	0.46 (0.40–0.52)	− 0.49–1.00	0.44 (0.41–0.47)	0.16–0.99	0.44 (0.41–0.47)	0.11–0.98
1	0.49 (0.40–0.58)	− 0.42–1.00	0.47 (0.43–0.51)	0.15–0.82	0.45 (0.41–0.49)	0.12–0.78
2	0.43 (0.28–0.58)	− 0.07–0.86	0.38 (0.32–0.44)	0.23–0.65	0.41 (0.34–0.48)	0.14–0.69
3	0.36 (− 0.46 to 1.00)	0.38 to 0.65	0.30 (0.08–0.52)	0.19–0.38	0.31 (0.01–0.61)	0.18–0.43

We visualized pairwise combination of the three instruments and examined the sensitivity of each by comparing the means and differences of HSU using Bland–Altman’s analysis (Fig. 2a–c). This method was used to show the agreement or bias between two paired quantitative measures [32]. Spearman’s correlation coefficient (Supplementary Table 4) was used to assess pairwise relationships between the instruments [10]. Spearman’s correlation coefficient is a non-parametric statistical measure used to test the strength and the direction of association between two ranked variables [33].

We examined floor and ceiling effects for the three MAUI (Table 4) Ceiling effects were assessed as HSU = 1.0 (full health). Floor effects were assessed as the worst patient-reported HSU for both the AQoL-8D and EQ-5D-5L-Psychosocial, and < 0 for the EQ-5D-5L (given that an HSU less than 0 is a health state deemed to be worse than death). We cross-matched against the individual HSU generated by the alternate instruments for individuals with ceiling and floor effects and then investigated the summary statistics for these HSU.

**Table 4:** Comparison of HSU floor and ceiling effects

MAUI (*N* = 198)				
	Floor		Ceiling	
	Mean (SD)	%	Mean (SD)	%
EQ-5D-5L	0.11 (0.20)	27 (13.6)	1.00 (0.00)	5 (2.5)
AQoL-8D	0.29 (0.09)	0	0.70 (0.26)	0
EQ-5D-5L-PSYCHOSOCIAL	0.25 (0.10)	0	0.74 (0.19)	0

We investigated summary HSU for each disability and fatigue severity classification. To validate our method, we expected that as disease and fatigue severity increased, mean and median HSU would decrease. We also investigated the proportions of responses for the four bolt-on dimensions of the EQ-5D-5L-Psychosocial across the five levels of dimensions (Table 5).

**Table 5:** Comparison of “Bolt-on dimensions” levels of responses

Bolt-on dimensions	Levels of responses *n* (%)									
	Participants with floor effect (* n* = 27)					Participants without floor effect (* n* = 171)				
	Worst response					Worst response				
	Level 1	Level 2	Level 3	Level 4	Level 5	Level 1	Level 2	Level 3	Level 4	Level 5
**Energy	0	0	0	3 (11)	24 (89)	0	2 (1)	27 (16)	56 (33)	86 (50)
Relationships	3 (11)	11 (41)	6 (22)	3 (11)	4 (15)	38 (22)	80 (47)	30 (18)	19 (11)	4 (2)
Sleeping	0	1 (4)	7 (26)	11 (41)	8 (30)	3 (2)	14 (8)	59 (35)	57 (33)	38 (22)
*Social solation	0	0	0	11 (41)	16 (59)	8 (5)	15 (9)	69 (40)	51 (30)	28 (16)

*Energy = Lack or reduced energy had the most effect in the two groups whilst a combination of Energy and Social isolation had the most effect in the group (*n* = 27) with floor effect

Univariable linear regression models were used to examine the association between mean HSU of the sociodemographic variables and disease classifications for the three MAUI (Table 6). A *p*-value of < 0.05 was assumed as statistically significant. STATA (version 17, Stata Corp), R Package and Microsoft Excel were used for statistical analyses.

**Table 6:** Univariable linear regression model

Univariable	EQ-5D-5L		AQoL-8D		EQ-5D-5L Psychosocial	
	*β* (95% CI)	*p*-value	*β* (95% CI)	*p*-value	*β* (95% CI)	*p*-value
Age group (years)						
< 45	Reference		Reference		Reference	
> 45 & ≤ 84	0.13 (0.04–0.22)*	< 0.01	0.04 (<− 0.01 to − 0.09)	0.07	0.05 (0.01–0.09)*	0.02
Gender						
Male	Reference		Reference		Reference	
Female	− 0.08 (− 0.20 to − 0.04)	0.18	− 0.03 (− 0.09 to − 0.03)	0.39	− 0.02 (− 0.08 to 0.04)	0.53
Education						
≤ Year 12	Reference		Reference		Reference	
Trade	− 0.05 (− 0.21 to − 0.11)	0.55	− 0.02 (− 0.10 to 0.05)	0.56	− 0.03 (− 0.10 to 0.05)	0.50
Bachelors	0.02 (− 0.12 to 0.17)	0.75	0.02 (− 0.06 to 0.09)	0.67	0.03 (− 0.04 to 0.10)	0.36
Postgraduate	0.07 (− 0.07 to 0.22)	0.33	0.06 (− 0.01 to 0.13)	0.11	0.06 (− 0.01 to − 0.13)	0.08
Employment						
Unwell	Reference		Reference		Reference	
Unemployed	0.06 (− 0.27 to 0.39)	0.72	0.06 (− 0.11 to 0.22)	0.49	0.02 (− 0.13 to 0.18)	0.76
Retired	0.24 (0.09 to 0.38)*	< 0.01	0.18 (0.11–0.25)*	< 0.01	0.16 (0.09–0.22)*	< 0.01
Full-time	0.30 (0.13 to 0.48)*	< 0.01	0.16 (0.07–0.24)*	< 0.01	0.15 (0.06–0.23)*	< 0.01
Part-time	0.11 (0.01 to 0.21)*	0.04	0.07 (0.02–0.12)*	0.01	0.05 (9.82e−06 to − 0.10)*	0.05
Student	0.13 (− 0.11 to 0.37)	0.28	0.05 (− 0.07 to 0.17)	0.39	0.02 (− 0.10 to 0.13)	0.78
Other	0.22 (− 0.02 to − 0.46)	0.07	0.06 (− 0.06 to − 0.17)	0.33	0.08 (− 0.03 to − 0.20)	0.14
Income						
Nil/negative income	Reference		Reference		Reference	
≤ 399/week	− 0.03 (− 0.18 to 0.12)	0.70	− 0.08 (− 0.15 to − 0.01)*	0.03	− 0.05 (− 0.13 to 0.02)	0.13
400–799/ week	− 0.03 (− 0.17 to 0.12)	0.73	− 0.04 (− 0.10 to 0.03)	0.31	− 0.02 (− 0.09 to 0.04)	0.47
800–1249/week	0.08 (− 0.11 to 0.28)	0.41	0.03 (− 0.06 to 0.12)	0.54	0.02 (− 0.07 to 0.12)	0.60
≥ 1250/week	0.18 (< − 0.01 to − 0.36)*	0.05	0.06 (− 0.03 to − 0.14)	0.20	0.05 (− 0.03 to 0.14)	0.24
Marital status						
Single	Reference		Reference		Reference	
Married	0.16 (0.07–0.26)*	< 0.01	0.06 (0.01–0.11)*	0.01	0.08 (0.03–0.12)*	< 0.01
Divorced/separated	0.11 (− 0.02 to 0.24)	0.10	0.02 (− 0.05 to 0.08)	0.59	0.03 (− 0.03 to 0.10)	0.29
Geographical Remoteness area						
Major city	Reference		Reference		Reference	
Inner regional	0.03 (− 0.07 to 0.14)	0.55	< 0.01 (− 0.05 to 0.06)	0.86	< 0.01 (− 0.05 to 0.05)	0.97
Outer regional	< − 0.01 (− 0.15 to 0.14)	0.96	0.01 (− 0.05 to 0.08)	0.67	< 0.01 (− 0.06 to 0.07)	0.94
Disability severity						
No	Reference		Reference		Reference	
Mild	− 0.31 (− 0.67 to 0.07)	0.11	− 0.42 (− 0.60 to − 0.24)*	< 0.01	− 0.39 (− 0.56 to − 0.22)*	< 0.01
Moderate	− 0.56 (− 0.93 to − 0.18)*	< 0.01	− 0.57 (− 0.75 to − 0.39)*	< 0.01	− 0.52 (− 0.68 to − 0.35)*	< 0.01
Severe	− 0.98 (− 1.37 to − 0.59)*	< 0.01	− 0.69 (− 0.88 to − 0.51)*	< − 0.01	− 0.70 (− 0.87 to − 0.52)*	< 0.01
Fatigue severity						
No	Reference		Reference		Reference	
Mild	− 0.31 (− 0.67 to − 0.06)	0.10	− 0.38 (− 0.56 to − 0.20)*	< 0.01	− 0.37 (− 0.54 to − 0.20)*	< 0.01
Moderate	− 0.45 (− 0.80 to − 0.10)*	0.01	− 0.54 (− 0.71 to − 0.36)*	< 0.01	− 0.47 (− 0.64 to − 0.31)*	< 0.01
Severe	− 0.84 (− 1.20 to − 0.49)*	< 0.01	− 0.65 (− 0.83 to − 0.48)*	< 0.01	− 0.63 (− 0.80 to − 0.47)*	< 0.01
Comorbidity						
0	Reference		Reference		Reference	
1	0.03 (− 0.08 to 0.14)	0.60	0.03 (− 0.02 to 0.08)	0.25	0.01 (− 0.04 to 0.06)	0.64
2	− 0.03 (− 0.19 to 0.13)	0.72	− 0.06 (− 0.14 to 0.01)	0.10	− 0.03 (− 0.10 to 0.05)	0.45
3	0.10 (− 0.42 to 0.22)	0.54	− 0.15 (− 0.30 to 0.01)	0.07	− 0.14 (− 0.29 to 0.01)	0.08

*Statistically significant in our model

## Results

### Participant characteristics

Overall, 201 participants attempted to complete the AQoL-8D and EQ-5D-5L, with 198 providing sufficient responses to calculate HSU. Non-completers and completers were similar across sociodemographic variables. Table 2 describes participants’ characteristics. The mean (SD) age was 48.7 years (14.3) with four-fifths being female (*n* = 158, 79.8%). Using the Australian Bureau of Statistics’ Statistical Geography Standard [34], over half resided in major cities (58.9%) and one-quarter in inner regional areas (28.1%). Almost half (43.9%) of the participants were either married/defacto whilst a third were single. Almost half (46.5%) said they were too unwell to work whilst one-quarter (26.7%) worked on a part-time basis. Of the participants with a source of income, two-fifths (40.5%) earned between $AUD400 and $AUD799 per week. The majority of participants reported no comorbid health conditions (64.7%), and one-quarter had at least one comorbidity (24.2%). For disability and fatigue severity, 69.2% reported moderate disability severity and 59.6% reported moderate fatigue severity respectively.

### EQ-5D-5L and AQoL-8D questionnaire completion

Of the 198 participants for whom we could generate an HSU using the EQ-5D-5L, we observed between 1 and 4 missing responses each in questions for the AQoL-8D relating to confidence (4), family relationship (4), mobility (4), cope with problems (4), help around house (6) and close relationships (3) respectively-(Supplementary Table 2). These missing values did not affect the utility values generated for the AQoL-8D.

### Summary HSU for sociodemographic and clinical characteristics, including disability and fatigue severity

Table 3 and Supplementary Table 3 summarizes HSU for the three MAUI. Mean HSU for the three instruments were similar: EQ-5D-5L 0.46 (95% CI 0.42–0.50); AQoL-8D 0.44 (95% CI 0.42–0.45); and EQ-5D-5L Psychosocial 0.44 (95% CI 0.42–0.46). Notably, these HSU were substantially reduced (almost half) compared to the general population norms (i.e. EQ-5D-5L: 0.89 and AQoL-8D: 0.77) [27, 28]. The median (IQR) HSU were similar for the EQ-5D-5L Psychosocial and AQoL-8D but higher for the EQ-5D-5L.

Mean HSU were higher in males compared to females across the three instruments and were observed to be highest amongst males for the EQ-5D-5L at 0.53 (95% CI 0.44–0.62). Participants in the < 45-year age category had lower mean HSU compared to the older age category (46–84 years) across all three instruments. The EQ-5D-5L recorded the highest mean HSU of 0.51 (0.45–0.57) for the older age category.

**Fig. 1:**
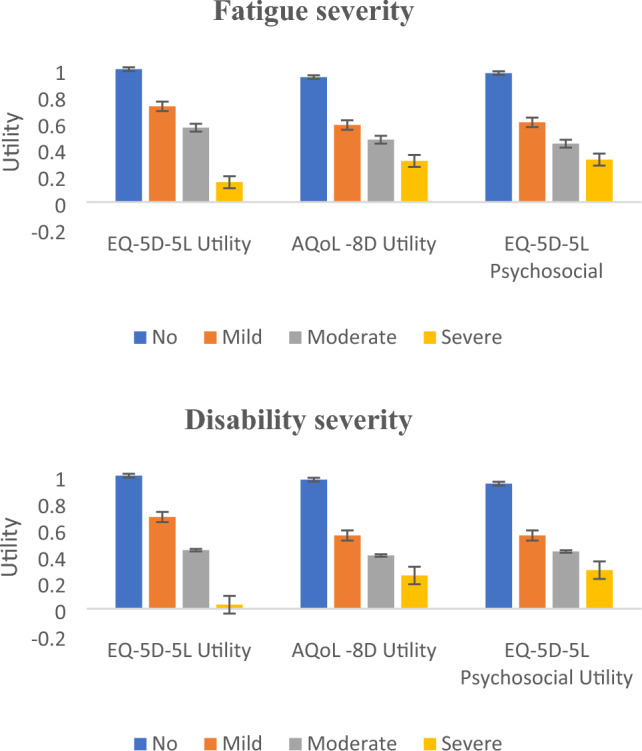
Mean HSUs based on disability and fatigue severity

Regarding the disability and fatigue severity classifications, mean HSU for all three instruments showed the expected inverse relationship: as disability severity and fatigue severity increased, the concomitant HSU decreased. Ideal health (1.00 for the EQ-5D-5L) and near-ideal health (0.99: AQoL-8D and 0.94: EQ-5D-5L-Psychosocial) for participants with no disability was observed. In addition, substantially diminished mean HSU for the severe disability severity classification of EQ-5D-5L (0.02), AQoL-8D (0.30) and EQ-5D-5L-Psychosocial (0.25) were reported. The substantially lower HSU generated from the EQ-5D-5L was due to its broader algorithmic range as noted in Table 1. Figure 1 shows the trend of mean HSU for disability severity and fatigue severity categories across the three MAUI.

### Instrument interchangeability

**Fig. 2:**
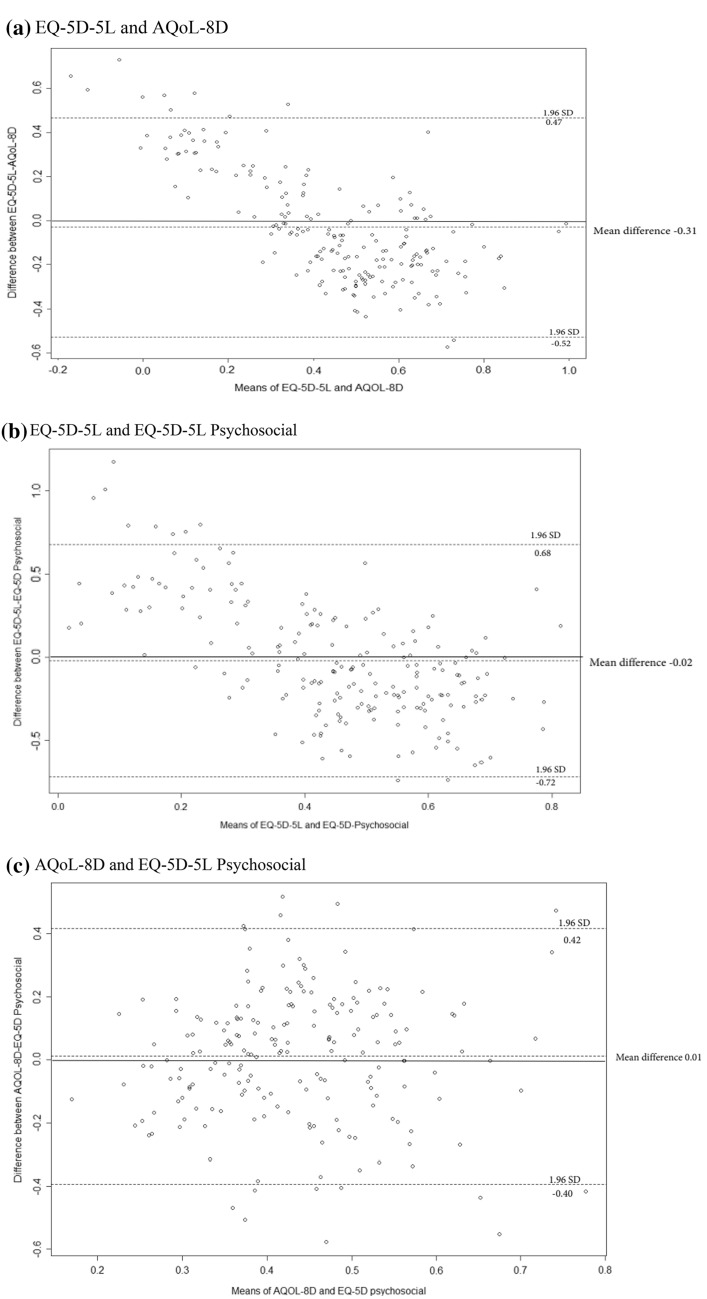
**a**–**c** Bland Altman’s Plots showing pair-wise differences and limits of agreements between instruments

Figure 2a–c show Bland Altman’s plots with positive mean differences between EQ-5D-5L and AQoL-8D = 0.03 (95% CI − 0.53 to 0.47); EQ-5D-5L and EQ-5D-5L-Pychosocial = 0.02 (95% CI − 0.72 to 0.68); and AQoL-8D and EQ-5D-5L-Psychosocial = 0.01 (95% CI − 0.40 to 0.42). The Spearman’s correlation coefficient for the three instruments was strong and positively correlated for the EQ-5D-5L-Psychosocial and the AQoL-8D (*ρ* 0.83; *p* < 0.0001) and the least positive correlation for the AQoL-8D and EQ-5D-5L (*ρ* 0.65; *p* < 0.0001). However, we note that a high correlation does not imply close agreement and is blind to the possibility of systematic bias [10, 32, 35]. To verify these results, we analysed Bland–Altman plots. Visualisation of the plots in Fig. 2a–c also revealed that there was systematic variation between the EQ-5D-5L and EQ-5D-5L-Psychosocial and the AQoL-8D and EQ-5D-5L. On the other hand, there was no evidence of systematic variation between the AQoL-8D and EQ-5D-5L-Psychosocial suggesting pairwise agreement for these two instruments, validating our results from the correlation analysis.

### Floor and ceiling effects

Table 4 shows the floor and ceiling effects for each instrument. For the EQ-5D-5L, a ceiling effect (i.e., participants reporting full/perfect health) was observed for 2.5% (*n* = 5) of participants, and a floor effect was observed for 13.6% (*n* = 27). In contrast, no floor or ceiling effects were observed for the EQ-5D-5L-Psychosocial or AQoL-8D instruments although both showed two high—but not perfect—utility valuations (EQ-5D-5L Psychosocial = 0.91 and 0.98); AQoL-8D (0.95 and 0.99).

To investigate the floor effect for the EQ-5D-5L in more detail, Table 5 shows a comparison of the responses to the bolt-on questions for the EQ-5D-5L-Psychosocial for the participants who reported a floor effect (*n* = 27) and those who did not (*n* = 171). We observed that 89% of participants in the *n* = 27 group always lacked energy whilst only 50% of the participants in *n* = 171 group felt the same way.

### Univariable regression analysis

Table 6 summarises the univariable regression models assessing the associations between mean HSU and clinical variables across the three instruments. The strongest effects were associated with disability severity and fatigue severity. Firstly, compared to no disability, moderate and severe disability were associated with substantial decreases in HSU across all three instruments (*p* < 0.01). Mild disability was associated with decreased HSU with the AQoL-8D and EQ-5D-5L psychosocial instruments respectively (*p* < 0.01), but not for the EQ-5D-5L.

Effects were also observed for age, employment, and marital status. For age, small associations between older ages (45–84 years) and higher HSU were observed for the EQ-5D-5L alone and with the psychosocial questions (*p* < 0.01 and *p* = 0.02 respectively). Unsurprisingly, compared to the employment category “too unwell to work”, being employed full- or part-time was associated with improved HSU for all three instruments. In addition, being retired was also associated with higher HSU (*p* < 0.01 for all three instruments). With regard to relationship status, being married was associated with higher HSU for each instrument (*p* ≤ 0.01).

## Discussion

To the best of our knowledge, our study is the first to generate and compare HSU for people living with ME/CFS in Australia using three MAUI. We have provided much-needed evidence regarding the impact of ME/CFS on HRQoL, as measured by the HSU. Importantly, our model showed that both disability and fatigue severity were significant predictors of diminished HSU. We also conducted an exploratory head-to-head comparison of the three MAUI to ascertain which is preferentially sensitive, especially when taking participant burden into account, for the ME/CFS cohort. We propose that the EQ-5D-5L-Psychosocial is the preferred MAUI based on limited participant burden.

Our study showed mean HSU of 0.46 (EQ-5D-5L); 0.44 (AQoL-8D) and 0.44 (EQ-5D-5L- Psychosocial) respectively. These HSU were approximately half the Australian population norms: 0.89 for the EQ-5D-5L [28] and 0.77 for the AQoL-8D [27]. Whilst it is important to note that the representativeness of our sample is not known, our results are consistent with published studies. A Danish study that used the EQ-5D-3L instrument reported significantly lower HSU for ME/CFS patients (0.47) than the population mean (0.85) [8]. Similarly, Viyas et al. also used the EQ-5D-3L and reported a much lower mean HSU of 0.36 compared to the UK representative population mean of 0.86 [16]. Whilst not reporting HSU, an Australian study using the SF-36 reported substantial impacts on the physical role and energy/fatigue domains (summary scores were not reported) [7].

Our study showed that HSU were higher in the older age group category of 46–84 years. In contrast, population norm data for HSU typically reduce with increasing age [28, 36]. These differences may be explained by the findings of a study which reported that younger adults with chronic conditions were more likely to report disability and poorer quality of life compared to their older counterparts [37]. In turn, younger people are likely to be more socially active and therefore more impacted by chronic diseases [37]. We stratified our sample by all the sociodemographic and clinical factors. Higher mean HSU were observed for males, for participants aged > 45 years and for participants with no or mild disability and fatigue. Similarly, in the univariable regression analyses, worse disability and fatigue severity were associated with poorer HSU. Positive associations between older ages, being employed and being married were also observed.

The association between disability and fatigue severity with decreasing HSU is consistent with existing studies on comparable chronic conditions such as MS [38, 39]. Our analysis of the HSU for the disability and fatigue classifications suggest that the psychosocial domains of health were the drivers of the consistently lower HSU for the EQ-5D-5L Psychosocial and AQoL-8D instruments. We observed a 2.5% (*n* = 5) ceiling effect for our study population for the EQ-5D-5L: much lower than reported in other studies for people with complex and chronic disease [10]. In turn, this indicates the reduced HRQoL for our study participants. Additionally, due to the broader (and negative) algorithmic range of the EQ-5D-5L, lower HSU for people in the severe disability and fatigue classifications were observed. This suggests that the EQ-5D-5L was more sensitive for the severe classifications. Our univariable regression model showed age, employment status (retired, full time and part time), marital status (married), disability and fatigue severity as statistically significant predictors of HSU but not so for comorbidities. This is in tandem with a previously published study in the UK where ME/CFS patients were more likely to become unemployed due to reduced physical functioning [5]. However, another study reported that having CFS and other chronic conditions were strong predictors of poorer health status [40]. These differences could be explained by our participant burden and willingness to participate in our study.

When comparing the three instruments, we observed a strong positive correlation between the AQoL-8D and the EQ-5D-5L Psychosocial. Given the preferential sensitivity for ME/CFS for these two instruments and the substantially reduced participant burden (i.e., 35 questions for the AQoL-8D versus 9 for the EQ-5D-5L Psychosocial), we recommend use of the EQ-5D-5L Psychosocial. However, it should be noted that the EQ-5D-5L will be more sensitive for people living with severe ME/CFS.

A major strength of this study was the use of an innovative approach with three MAUI to generate HSU for our study population for the first time. This has allowed us to identify an instrument that is preferentially sensitive for ME/CFS and, importantly, minimises participant burden. Another strength is the collaboration between the researchers and the Patient Advisory Group, and development of the survey based on evidence from the focus groups. Together we identified the need to include participants experiencing a range of disease severity states. In addition, we developed discrete modules in the online survey to support participant’s survey completion at their own pace. This resulted in the development of a comprehensive survey that did not impose significant cognitive burden on participants. Another strength is that we used a validated method to classify disability and fatigue severity scores to investigate the HSU for these classifications to assess instrument sensitivity.

Our study has three major limitations. First, we used convenience sampling, so we cannot comment on the representativeness of our study population. As a result, extrapolating our findings to the larger ME/CFS population should be done with caution. We aimed to address this limitation by categorising HSU based on disease and fatigue severity. Second, because there is no published Australian value set for the EQ-5D-5L, we calculated HSU using an Australian algorithm based on a discrete choice experiment. Third, we relied on participants' self-reports of ME/CFS diagnoses rather than physician confirmation, which may have introduced bias into this study.

## Conclusion

ME/CFS has a profound impact on people’s HRQoL regardless of their age and gender. Across the three MAUI, mean HSU ranged from 0.44 to 0.46 reflecting the extent of this impact. Importantly, this is approximately half the Australian population norms. Our exploratory head-to-head comparison results revealed that the psychosocial bolt-ons to the EQ-5D-5L were important domains of health for people living with ME/CFS. Moreover, our study also revealed the interchangeability of the AQoL-8D and EQ-5D-5L-Psychosocial instruments. Given participant burden and the importance of the psychosocial bolt-ons for ME/CFS, the EQ-5D-5L-Psychosocial is recommended as a preferred MAUI for people living with ME/CFS.

### Supplementary Information

Below is the link to the electronic supplementary material.

Supplementary file1 (DOCX 167 KB)

## Data Availability

The participants of this study did not give written consent for their data to be shared publicly, so due to the sensitive nature of the research supporting data is not available.
