# Supplementary material for: Assessing health state utilities for people with myalgic encephalomyelitis/chronic fatigue syndrome in Australia using the EQ-5D-5L, AQoL-8D and EQ-5D-5L-psychosocial instruments
Source: Qual Life Res. 2023 Aug 10;33(1):45–57. doi: 10.1007/s11136-023-03498-8 (PMC10784392; doi:10.1007/s11136-023-03498-8)
Supplement: Supplementary file 1 — Supplementary file1 (DOCX 167 KB) [file 11136_2023_3498_MOESM1_ESM.docx]

**Supplementary Figure 1:**

Flow of participants into the study with the generation of participant health state utilities (HSU) for the EQ-5D-5L, AQoL-8D and EQ-5D-5L-Psychosocial multi-attribute utility instruments.


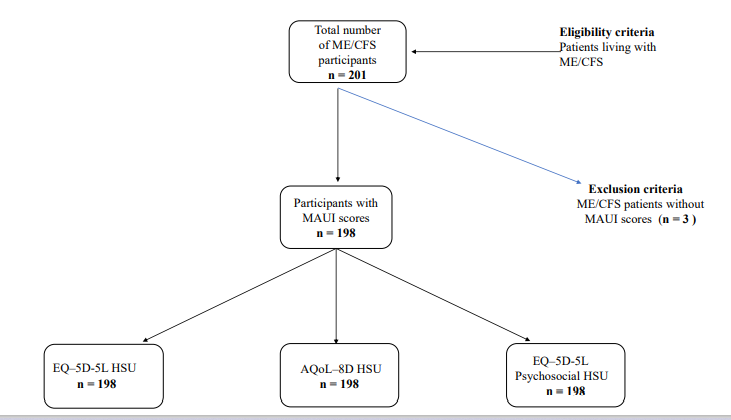


**Supplementary Table 1: Fatigue severity-related questions**

|  | 1. Fatigue | |
| --- | --- | --- |
| 1. | Q36 *Frequency:*   - None of the time (1) - A little of the time (2) - About half of the time (3) - Most of the time (4) - All of the time (5) | Q37 *Severity:*   - Symptom not present (1) - Mild (2) - Moderate (3) - Severe (4) - Very severe (5) |
| 2. | **Next-day soreness after non-strenuous activities** | |
|  | Q38 *Frequency:*   - None of the time (1) - A little of the time (2) - About half of the time (3) - Most of the time (4) - All of the time (5) | Q39 *Severity:*   - Symptom not present (1) - Mild (2) - Moderate (3) - Severe (4) - Very severe (5) |
| 3 | **Minimum exercise makes you physically tired** | |
|  | Q40*.Frequency:*   - None of the time (1) - A little of the time (2) - About half of the time (3) - Most of the time (4) - All of the time (5) | Q41 *Severity:*   - Symptom not present (1) - Mild (2) - Moderate (3) - Severe (4) - Very severe (5) |
| 4 | **Feeling unrefreshed after you wake up in the morning** | |
|  | Q42. *Frequency:*   - None of the time (1) - A little of the time (2) - About half of the time (3) - Most of the time (4) - All of the time (5) | Q43 *Severity:*   - Symptom not present (1) - Mild (2) - Moderate (3) - Severe (4) - Very severe (5) |
| 5. | **Problems remembering things** | |
|  | Q50. *Frequency:*   - None of the time (1) - A little of the time (2) - About half of the time (3) - Most of the time (4) - All of the time (5) | Q51 *Severity:*   - Symptom not present (1) - Mild (2) - Moderate (3) - Severe (4) - Very severe (5) |
| 6 | **Difficulty paying attention for a long period of time** | |
|  | Q52 *Frequency:*   - None of the time (1) - A little of the time (2) - About half of the time (3) - Most of the time (4) - All of the time (5) | Q53 *Severity:*   - Symptom not present (1) - Mild (2) - Moderate (3) - Severe (4) - Very severe (5) |
| 7 | **Feeling unsteady on your feet, like you might fall** | |
|  | Q56. *Frequency:*   - None of the time (1) - A little of the time (2) - About half of the time (3) - Most of the time (4) - All of the time (5) | Q57 *Severity:*   - Symptom not present (1) - Mild (2) - Moderate (3) - Severe (4) - Very severe (5) |

| **Supplementary Table 2: Questionnaire completion** Missing data points for the items of the EQ-5D-5L and AQoL-8D. | | | | | | | | |
| --- | --- | --- | --- | --- | --- | --- | --- | --- |
| **EQ-5D-5L** | | | |  |  |  |  |  |
| S/N | Item | Missing | Available |  |  |  |  |  |
|  |  | (n=x) (n=x) | |  |  |  |  |  |
| 1 | *Mobility* | 3 | 198 |  |  |  |  |  |
| 2 | *Self-care* | 3 | 198 |  |  |  |  |  |
| 3 | *Usual activities* | 3 | 198 |  |  |  |  |  |
| 4 | *Pain* | 3 | 198 |  |  |  |  |  |
| 5 | *Anxiety and depression* | 3 | 198 |  |  |  |  |  |
| 6 | Health State Utility | 3 | 198 |  |  |  |  |  |
|  |  |  |  |  |  |  |  |  |
| **AQoL -8D** | | | |  |  |  |  |  |
| S/N | Item | Missing | Available |  |  |  |  |  |
|  |  | (n=x) (n=x) | |  |  |  |  |  |
| 1 | *Energy* | 3 | 198 |  |  |  |  |  |
| 2 | *Socially Excluded* | 3 | 198 |  |  |  |  |  |
| 3 | *Getting Around* | 3 | 198 |  |  |  |  |  |
| 4 | *Role in Community* | 5 | 196 |  |  |  |  |  |
| 5 | *Feel Sad* | 3 | 198 |  |  |  |  |  |
| 6 | *Serious Pain* | 3 | 198 |  |  |  |  |  |
| 7 | *Confidence* | 4 | 197 |  |  |  |  |  |
| 8 | *Calm* | 3 | 198 |  |  |  |  |  |
| 9 | *Family Relationships* | 4 | 197 |  |  |  |  |  |
| 10 | *Close Relationships* | 3 | 198 |  |  |  |  |  |
| 11 | *Communication* | 3 | 198 |  |  |  |  |  |
| 12 | *Trouble Sleeping* | 3 | 198 |  |  |  |  |  |
| 13 | *Worthless* | 3 | 198 |  |  |  |  |  |
| 14 | *Angry* | 3 | 198 |  |  |  |  |  |
| 15 | *Mobility* | 4 | 197 |  |  |  |  |  |
| 16 | *Hurting Yourself* | 3 | 198 |  |  |  |  |  |
| 17 | *Enthusiastic* | 3 | 198 |  |  |  |  |  |
| 18 | *Feel Worried* | 3 | 198 |  |  |  |  |  |
| 19 | *Washing* | 3 | 198 |  |  |  |  |  |
| 20 | *Feel Happy* | 3 | 198 |  |  |  |  |  |
| 21 | *Cope With Problems* | 4 | 197 |  |  |  |  |  |
| 22 | *Pain* | 3 | 198 |  |  |  |  |  |
| 23 | *Enjoy Relationships* | 3 | 198 |  |  |  |  |  |
| 24 | *Pain Interfere* | 3 | 198 |  |  |  |  |  |
| 25 | *Feel Pleasure* | 3 | 198 |  |  |  |  |  |
| 26 | *Burden On Others* | 3 | 198 |  |  |  |  |  |
| 27 | *Content* | 3 | 198 |  |  |  |  |  |
| 28 | *Vision* | 3 | 198 |  |  |  |  |  |
| 29 | *In Control of Life* | 3 | 198 |  |  |  |  |  |
| 30 | *Help Around House* | 6 | 195 |  |  |  |  |  |
| 31 | *Feel Isolated* | 3 | 198 |  |  |  |  |  |
| 32 | *Hearing* | 3 | 198 |  |  |  |  |  |
| 33 | *Depressed* | 3 | 198 |  |  |  |  |  |
| 34 | *Close Relations Happy* | 6 | 195 |  |  |  |  |  |
| 35 | *Feel Despair* | 3 | 198 |  |  |  |  |  |
| 36 | Health State Utility | 3 | 198 |  |  |  |  |  |

**Supplementary Table 3: Median and IQR HSUs for the EQ-5D-5L, AQoL-8D and EQ-5D-5L-Psychosocial Multi-attribute Instruments**

|  | **EQ-5D-5L** | | **AQoL-8D** |  | **EQ-5D-5L- Psychometric** | |
| --- | --- | --- | --- | --- | --- | --- |
|  | **Median (IQR)** | **n (%)** | **Median (IQR)** | **n (%)** | **Median (IQR)** | **Median (IQR)** |
| ***Overall sample*** | 0.52 (0.30 - 0.67) | 198 (100) | 0.41 (0.34 - 0.54) | 198 (100) | 0.43 (0.34 - 0.56) | 198 (100) |
| ***Sociodemographic*** |  |  |  |  |  |  |
| *Age group (years)* |  |  |  |  |  |  |
| <45 | 0.40 (0.12 - 0.64) | 73 (37.1) | 0.40(0.32 - 0.50) | 73 (37.1) | 0.38(0.31 - 0.52) | 73(37.1) |
| 46-84 | 0.58 (0.38 - 0.70) | 124(62.9) | 0.43(0.35 - 0.55) | 124 (62.9) | 0.45(0.35 - 0.56) | 124(62.9) |
| ***Gender*** |  |  |  |  |  |  |
| Male | 0.59(0.39 - 0.68) | 32(16.2) | 0.44(0.32 - 0.57) | 32 (16.2) | 0.48(0.35 - 0.56) | 32(16.2) |
| Female | 0.52(0.27 - 0.67) | 158(79.8) | 0.41(0.35 - 0.54) | 158 (79.8) | 0.44(0.34 - 0.56) | 158(79.8) |
| Other | 0.49(0.09 - 0.63) | 8(4.0) | 0.37(0.29 - 0.46) | 8 (4.0) | 0.38(0.27 - 0.49) | 8(4.0) |
| ***Education Level*** |  |  |  |  |  |  |
| ≤ Year 12 | 0.58(0.28 - 0.67) | 25(12.7) | 0.38(0.31 - 0.53) | 25(12.7) | 0.38(0.28 - 0.51) | 25(12.7) |
| Trade | 0.49(0.12 - 0.66) | 40(20.3) | 0.39(0.29 - 0.49) | 40(20.3) | 0.39(0.27 - 0.53) | 40(20.3) |
| Bachelors | 0.52(0.32 - 0.67) | 65(33.0) | 0.40(0.35 - 0.52) | 65(33.0) | 0.43(0.35 - 0.56) | 65(33.0) |
| Postgraduate | 0.59(0.38 - 0.73) | 67(34.0) | 0.46(0.35 - 0.59) | 67(34.0) | 0.48(0.38 - 0.58) | 67(34.0) |
| ***Employment*** |  |  |  |  |  |  |
| Unwell | 0.48(0.12 - 0.66) | 80(46.5) | 0.38(0.32 - 0.47) | 80(46.5) | 0.39(0.33 - 0.50) | 80(46.5) |
| Unemployed | 0.39(0.34 – 0.64) | 3(1.7) | 0.40(0.28 - 0.68) | 3(1.7) | 0.40(0.33 - 0.56) | 3(1.7) |
| Retired | 0.67(0.56 - 0.79) | 19(11.1) | 0.60(0.42 - 0.74) | 19(11.1) | 0.56(0.46 - 0.69) | 19(11.1) |
| Full-time | 0.74(0.54 - 0.83) | 12(7.0) | 0.56(0.46 - 0.66) | 12(7.0) | 0.56(0.46 - 0.65) | 12(7.0) |
| Part-time | 0.56(0.38 - 0.67) | 46(26.7) | 0.43(0.36 - 0.54) | 46(26.7) | 0.44(0.37 - 0.56) | 46(26.7) |
| Student | 0.49(0.33 - 0.72) | 6(3.5) | 0.44(0.34 - 0.54) | 6(3.5) | 0.43(0.31 - 0.45) | 6(3.5) |
| Other | 0.66(0.56 - 0.67) | 6(3.5) | 0.43(0.41 - 0.55) | 6(3.5) | 0.51(0.49 - 0.53) | 6(3.5) |
| ***Income*** |  |  |  |  |  |  |
| Nil/Negative income | 0.53(0.19 - 0.64) | 25(12.8) | 0.38(0.32 - 0.60) | 25(12.8) | 0.39(0.33 - 0.56) | 25(12.8) |
| ≤399/week | 0.51(0.14 - 0.67) | 51(26.2) | 0.37(0.29 - 0.46) | 51(26.2) | 0.40(0.28 - 0.52) | 51(26.2) |
| 400-799/week | 0.49(0.27 - 0.67) | 79(40.5) | 0.42(0.33 - 0.53) | 79(40.5) | 0.43(0.34 - 0.56) | 79(40.5) |
| 800 -1249/week | 0.58(0.40 - 0.67) | 17 (8.7) | 0.41(0.36 - 0.66) | 17 (8.7) | 0.46(0.39 - 0.52) | 17 (8.7) |
| ≥1250/week | 0.65(0.51 - 0.74) | 23(11.8) | 0.48(0.41 - 0.67) | 23(11.8) | 0.51(0.40 - 0.64) | 23(11.8) |
| ***Marital status*** |  |  |  |  |  |  |
| Single | 0.40(0.12 - 0.65) | 77(38.9) | 0.39(0.32 - 0.49) | 77(38.9) | 0.39(0.32 - 0.49) | 77(38.9) |
| Married | 0.59(0.39 - 0.73) | 87(43.9) | 0.43(0.35 - 0.60) | 87(43.9) | 0.50(0.37 - 0.59) | 87(43.9) |
| Divorced/Separated | 0.57(0.32 - 0.67) | 30(15.2) | 0.40(0.35 - 0.53) | 30(15.2) | 0.44(0.31 - 0.56) | 30(15.2) |
| Widowed | 0.68(0.53 - 0.85) | n/r* | 0.58(0.41 - 0.86) | n/r* | 0.55(0.43 - 0.78) | n/r* |
| ***Geographical remotenes****s* ***area*** |  |  |  |  |  |  |
| Major city | 0.53(0.32 - 0.67) | 113(58.9) | 0.41(0.35 - 0.53) | 113(58.9) | 0.43(0.34 - 0.54) | 113(58.9) |
| Inner regional | 0.59(0.31 - 0.72) | 54(28.1) | 0.40(0.32 - 0.55) | 54(28.1) | 0.45(0.33 - 0.56) | 54(28.1) |
| Outer regional | 0.52(0.24 - 0.66) | 25(13.0) | 0.45(0.36 – 0.55) | 23(13.0) | 0.43(0.36 - 0.58) | 23(13.0) |
| ***Clinical*** |  |  |  |  |  |  |
| ***Disability severity*** |  |  |  |  |  |  |
| No | 1(1.00 -1.00) | n/r* | 0.99(0.99 - 0.99) | n/r* | 0.94(0.91 - 0.98) | n/r* |
| Mild | 0.73(0.61 - 0.84) | 40 (20.2) | 0.55(0.46 - 0.68) | 40(20.2) | 0.56(0.50 - 0.64) | 40(20.2) |
| Moderate | 0.50(0.30 - 0.66) | 137 (69.2) | 0.39(0.34 - 0.49) | 137(69.2) | 0.43(0.34-0.52) | 137(69.2) |
| Severe | < -0.01(- 0.18 - 0.21) | 19 (9.6) | 0.31(0.20 -0.38) | 19(9.6) | 0.21(0.16 - 0.36) | 19(9.6) |
| **Fatigue severity** |  |  |  |  |  |  |
| No | 1.00 (1.00 - 1.00) | n/r* | 0.99(0.99 -0.99) | n/r* | 0.94(0.91 – 0.98) | n/r* |
| Mild | 0.75(0.58 - 0.86) | 22(11.1) | 0.63(0.52 - 0.70) | 22(11.1) | 0.57(0.50 – 0.66) | 22(11.1) |
| Moderate | 0.63(0.47 - 0.71) | 118(59.6) | 0.44(0.36 - 0.55) | 118(59.6) | 0.47(0.37 – 0.56) | 118(59.6) |
| Severe | 0.11(-0.07 - 0.39) | 56(28.3) | 0.32(0.27 - 0.39) | 56(28.3) | 0.31(0.23 – 0.39) | 56(28.3) |
| **Comorbidities** |  |  |  |  |  |  |
| 0 | 0.52(0.31 - 0.67) | 128(64.7) | 0.41(0.34 - 0.54) | 128(64.7) | 0.44(0.34 - 0.56) | 128(64.7) |
| 1 | 0.61(0.28 - 0.70) | 48(24.2) | 0.45(0.39 - 0.58) | 48(24.2) | 0.48(0.35 - 0.56) | 48(24.2) |
| 2 | 0.50(0.21 - 0.70) | 18(9.1) | 0.36(0.29 - 0.43) | 18(9.1) | 0.43(0.34 - 0.48) | 18(9.1) |
| 3 | 0.34(0.14 - 0.58) | n/r* | 0.32(0.23 - 0.36) | n/r* | 0.30(0.20 - 0.41) | n/r* |

**Supplementary Table 4: Spearman’s Correlation Coefficient**

| MAU Instruments | **EQoL-5D** | AQoL-8D | EQoL-5D-5L Pysc |
| --- | --- | --- | --- |
| EQoL-5D | 1 |  |  |
| AQoL-8D | 0.654 | 1 |  |
| EQoL-5D-5L Pysch | 0.826 | 0.826 | 1 |

**Supplementary Figure 2**: Histograms showing distribution of individual HSUs for the EQ-5D-5L, AQoL-8D and EQ-5D-5L-Psychosocial.

| **EQ-5D-5L HSU** | **AQoL-8D HSU** | **EQ-5D-5L Psychosocial HSU** |
| --- | --- | --- |
| 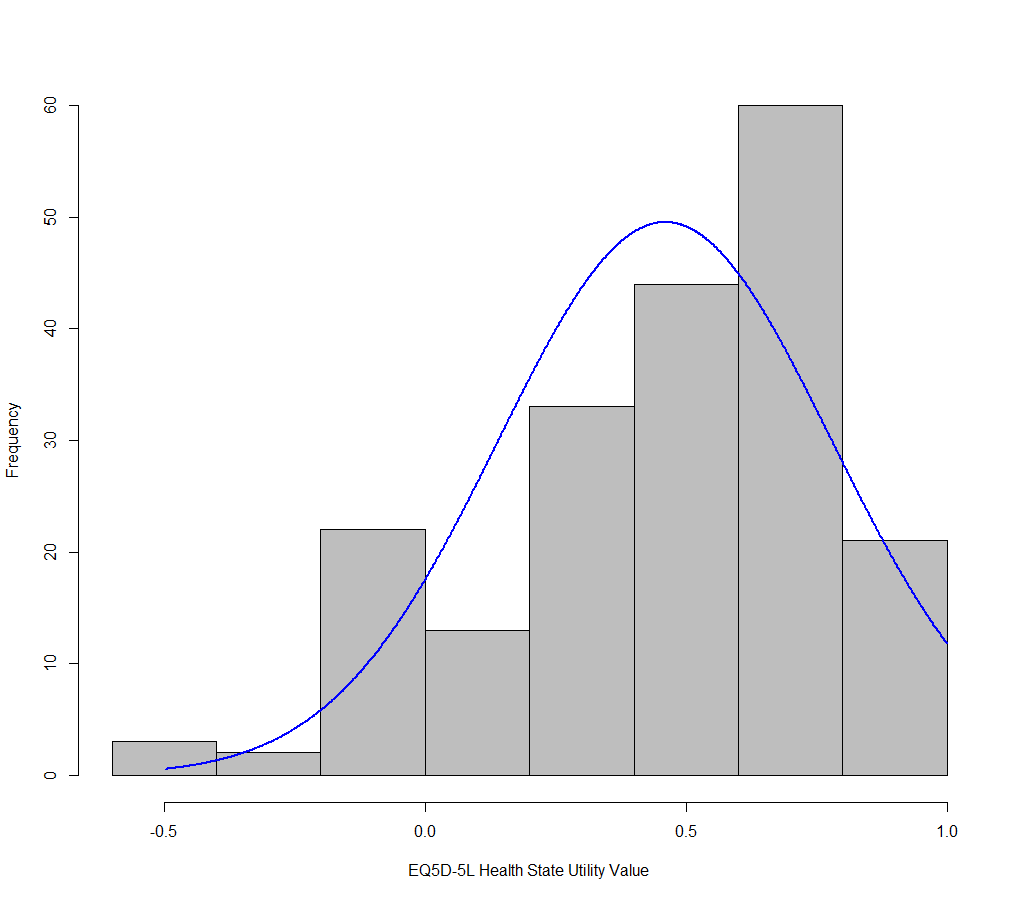 | 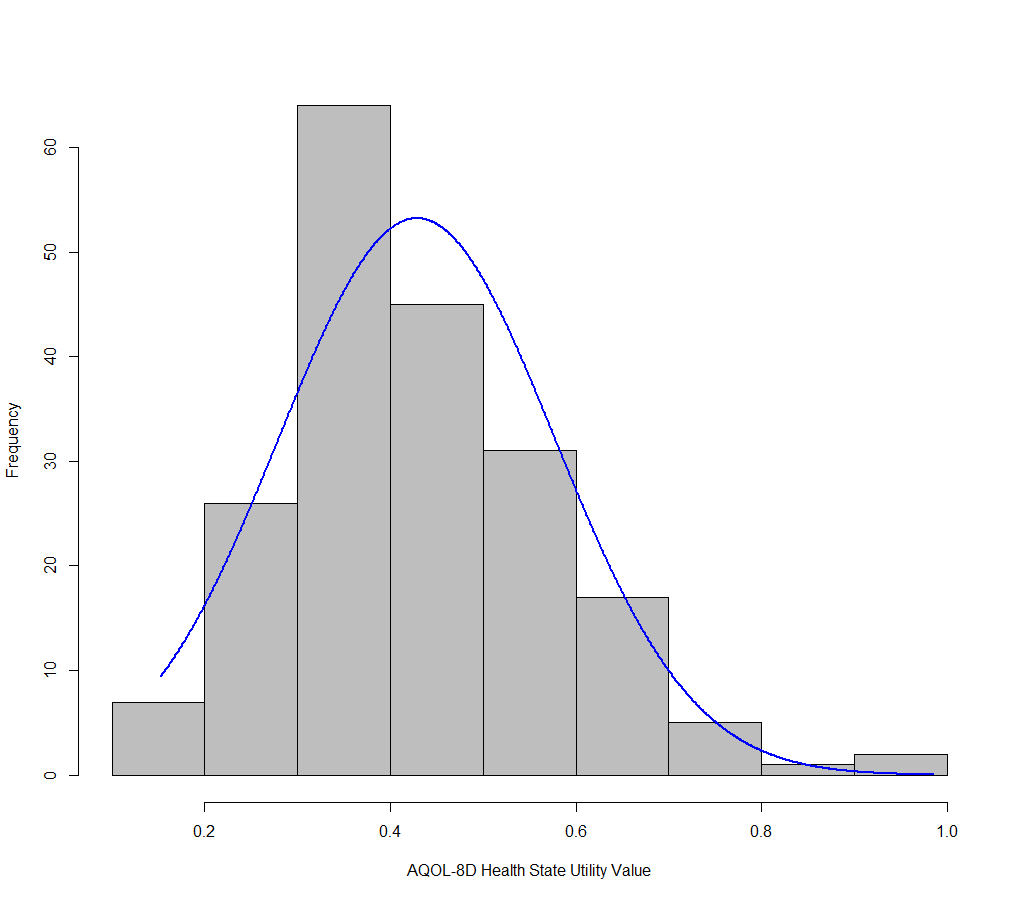 | 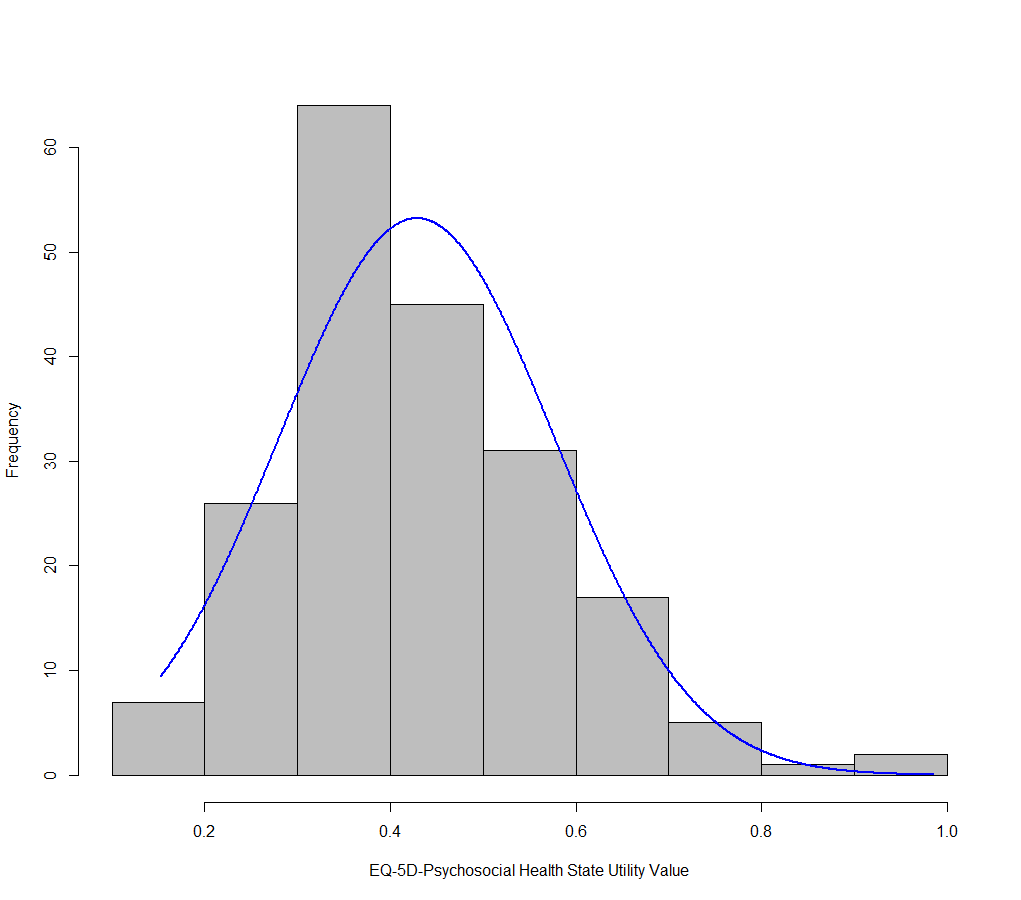 |
